# Elevated Ratio of Th17 Cell-Derived Th1 Cells (CD161^+^Th1 Cells) to CD161^+^Th17 Cells in Peripheral Blood of Early-Onset Rheumatoid Arthritis Patients

**DOI:** 10.1155/2016/4186027

**Published:** 2016-03-30

**Authors:** Shigeru Kotake, Yuki Nanke, Toru Yago, Manabu Kawamoto, Tsuyoshi Kobashigawa, Hisashi Yamanaka

**Affiliations:** Institute of Rheumatology, Tokyo Women's Medical University, 10-22 Kawada-cho, Shinjuku, Tokyo 162-0054, Japan

## Abstract

Rheumatoid arthritis (RA) is a chronic inflammatory disease characterized by the destruction of articular cartilage and bone with elevated levels of proinflammatory cytokines. It has been reported that IL-17 and Th17 cells play important roles in the pathogenesis of RA. Recently, plasticity in helper T cells has been demonstrated; Th17 cells can convert to Th1 cells. It remains to be elucidated whether this conversion occurs in the early phase of RA. Here, we tried to identify Th17 cells, Th1 cells, and Th17 cell-derived Th1 cells (CD161^+^Th1 cells) in the peripheral blood of early-onset RA patients. We also evaluated the effect of methotrexate on the ratio of Th17 cells in early-onset RA patients. The ratio of Th17 cell-derived Th1 cells to CD161^+^Th17 cells was elevated in the peripheral blood of early-onset RA patients. In addition, MTX reduced the ratio of Th17 cells but not Th1 cells. These findings suggest that IL-17 and Th17 play important roles in the early phase of RA; thus, anti-IL-17 antibodies should be administered to patients with RA in the early phase.

## 1. Introduction

Rheumatoid arthritis (RA) is a chronic inflammatory disease characterized by the destruction of articular cartilage and bone with elevated levels of proinflammatory cytokines, such as TNF*α* and IL-6, produced from the synovial tissue [1]. We previously reported that IL-17 from activated human T cells in the synovial tissues of patients with rheumatoid arthritis (RA) is a potent stimulator of osteoclast formation [2]. In 2005, human helper T-17 type cells (Th17 cells) were identified as helper T cells, distinct from Th1 or Th2 cells [3]. Since this identification of Th17 cells, it has been reported that they play important roles in the pathogenesis of RA [4, 5].

Several reports confirm that IL-17 is an important cytokine in the early phase or the disease-onset phase of RA. In 2005, Raza et al. reported that the peripheral level of IL-17 is significantly high, analyzing the patients with RA whose disease durations were less than nine weeks [6]. Kokkonen et al. reported that the concentration of IL-17 in individuals before disease onset is significantly higher than that in patients after disease onset [7]. In addition, Kochi et al. [8] demonstrated that a regulatory variant in CCR6, which is a specific marker for Th17 cells distinguishing them from other helper T cells [9, 10], is associated with RA susceptibility. The CCR6 dinucleotide polymorphism genotype is correlated with the expression level of CCR6 and is associated with the presence of IL-17 in the sera of subjects with RA [8]. Thus, it is speculated that IL-17 plays an important role in the disease-onset or the early phase of RA.

Recently, plasticity in helper T cells has been demonstrated [11]. It has been reported that Th17 cells can convert to Th1 cells [12]. In 2008, Cosmi et al. reported that CD161 is a marker of human Th17 cells [13]. In addition, Th17 cell-derived Th1 cells express CD161, which is detected in the synovial fluid from patients with juvenile idiopathic arthritis; thus, these cells are clearly distinct from Th1 cells [14–16]. Th17 cell-derived Th1 cells are also named “non-classic Th1 cells” [16]. In contrast, Th1 cells rather than Th17 cells were reported to be predominant in the peripheral blood of patients with late phase of RA whose average disease duration was 13 years [17]. We hypothesized that Th17 cells convert to Th1 cells in the early phase of RA and that methotrexate has an effect on the ratio of peripheral Th cells.

In the current study, we first evaluated the effect of methotrexate (MTX) on the ratio of Th cells in early-onset RA patients and then tried to identify Th17 cells, Th1 cells, and Th17 cell-derived Th1 cells in the peripheral blood of these early-onset RA patients. We report that MTX reduced the ratio of Th17 cells but not Th1 cells and that the ratio of Th17 cell-derived Th1 cells to Th17 cells was elevated in peripheral blood of early-onset RA patients.

## 2. Patients and Methods

### 2.1. Profiles of Patients

We analyzed two groups of patients with early-onset rheumatoid arthritis (RA). The RA patients met the ACR 1987 revised classification criteria. The 1st group comprised 5 patients (4 females and 1 male) whose disease durations were less than 18 months (Table 1). All patients were treated with methotrexate (MTX). The duration between first and second analysis was 1 to 6 months. RA patients were not treated by DMARDs or corticosteroids when peripheral blood was obtained. The peripheral helper T cells of these patients were analyzed according to the expressions of cytokines, interferon-*γ* (IFN-*γ*) and interleukin-17 (IL-17).

The 2nd group of patients comprised 6 patients (5 RA and 1 reactive arthritis [ReA]) (Table 2). ReA was a* Chlamydia*-associated arthritis. All patients were female. Six female, age-matched osteoarthritis (OA) patients were also analyzed as controls (data not shown). The disease durations of RA patients were less than 5 months. RA and ReA patients were not treated by DMARDs or corticosteroids when peripheral blood was obtained. The peripheral helper T cells of these RA, ReA, and OA patients were analyzed according to the expression of both CD161 and cytokines, interferon-*γ* (IFN-*γ*) and interleukin-17 (IL-17).

The current study was approved by the ethical committee of Tokyo Women's Medical University. Informed consent was obtained from each patient.

### 2.2. Flow Cytometry Analysis for CD4, CD161, and Intracellular IFN-*γ* and IL-17

After separating peripheral blood mononuclear cells (PBMCs), these cells were stimulated with 25 ng/mL PMA (Sigma) and 2 *μ*g/mL ionomycin (Sigma) in the presence of 10 mg/mL brefeldin-A (BFA, Sigma) for 4 h at 37°C in 7% CO_2_. T cells (400 *μ*L) were incubated with 2 mL of 1x FACS lysing solution (Becton Dickinson, Mountain View, CA) for 10 min at room temperature. PBMCs were washed and incubated with 500 *μ*L of 1x FACS permeabilizing solution (Becton Dickinson) for 10 min at room temperature. PBMCs were washed again and further incubated with PC5-conjugated anti-CD4 antibodies (Beckman Coulter), FITC-conjugated anti-human IFN-*γ* antibodies (Becton Dickinson), and Alexa Fluor 647-conjugated anti-human IL-17 antibodies (BD Bioscience) for 30 min at room temperature in the dark. In the analysis of the 2nd group of RA patients, PE-conjugated anti-CD161 antibodies (Becton Dickinson) were added to this incubation. The stained cells were analyzed using FACScan (BD Bioscience).

### 2.3. Statistical Analysis

Data were analyzed using the Wilcoxon signed-rank test and Mann-Whitney's *U* test (StatView®; Abacus Concepts Inc., Berkeley, CA). Data are presented as the mean ± SD. Significant difference was defined as *p* < 0.05.

## 3. Results

### 3.1. MTX Significantly Reduced the Ratio of Th17 Cells to Th Cells, but Not Those of Th1 Cells, “Both IFN-*γ* and IL-17 Positive Cells” (“Th17∙Th1 Cells”)

**Figure 1:**
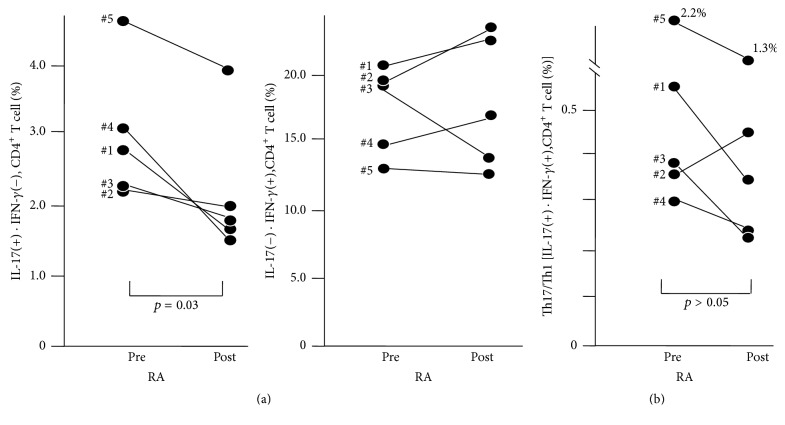
(a) Effect of MTX on the ratio of Th17 cells (left) or Th1 cells (right) to helper T cells. Number by the dot shows the patient number in Table 1. (b) Effect of MTX on the ratio of Th1-Th17 cells to helper T cells. Number by the dot shows the patient number in Table 1.

In the current study, we identified CD4^+^ cells as Th cells, IL-17^+^∙IFN-*γ* ^−^ CD4^+^ T cells as Th17 cells, IL-17^−^∙IFN-*γ* ^+^ CD4^+^ T cells as Th1 cells, and both positive IL-17^+^∙IFN-*γ* ^+^ CD4^+^ T cells as “Th17∙Th1 cells.” [We do not use “Th17/Th1 cells” because the expression may be confused with the ratio of Th1 to Th17.] In the 1st group of RA patients, the ratio of Th17 cells to helper T cells (Th cells) was significantly reduced by MTX treatment (*p* = 0.03, Figure 1(a) left). Th1 cells were not reduced by MTX treatment (Figure 1(a) right); the ratio increased in 3 out of 5 patients. The ratio of Th1∙Th17 cells was not reduced by MTX treatment (Figure 1(b)).

### 3.2. The Ratio of Th17 Cells to Helper T Cells

**Figure 2:**
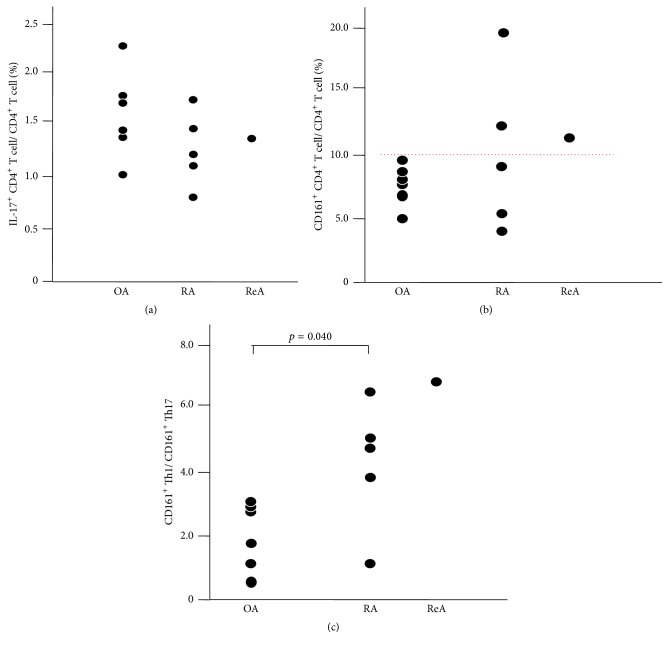
(a) Ratio of Th17 cells to helper T cells. (b) Ratio of CD161^+^ helper T cells to helper T cells. The red dotted line shows the highest ratio of OA patients. (c) Ratio of CD161^+^Th1 cells to CD161^+^Th17 cells.

Figure 2(a) shows the ratio of Th17 cells to Th cells in the 2nd group of RA and reactive arthritis (ReA) patients. There was no significant difference among OA, RA, and ReA.

### 3.3. The Ratio of CD161^+^ Helper T Cells to Helper T Cells

CD161 has been reported as a marker of human Th17 cells [13]; however, Th1 cells derived from Th17 cells also express CD161 [14]. We examined the ratio of CD161^+^CD4^+^ T cells to CD4^+^ T cells (Figure 2(b)). There was a tendency for the ratio to be higher in RA and ReA than in OA; the ratios in 3 of 6 patients with RA or ReA were higher than the highest ratio in OA patients (a red dotted line) although the difference was not statistically significant.

### 3.4. The Ratio of CD161^+^Th1 Cells to CD161^+^Th17 Cells

We then examined the ratio of CD161^+^Th1 cells to CD161^+^Th17 cells (Figure 2(c)). The ratio of RA was significantly higher than that of OA (*p* = 0.04, Figure 2(c)). The ratio of ReA was highest among all of the data.

## 4. Discussion

In the current study, we clearly demonstrated that in the early-onset RA patients MTX reduced the ratio of Th17 cells among helper T cells but not those of Th1 cells or Th17∙Th1 cells (Th cells producing both IFN-*γ* and IL-17). In addition, we also showed that the ratio of Th17 cell-derived Th1 cells to Th17 cells increased in the peripheral blood of the early-onset RA patients, compared with those of OA patients.

It has been reported that the anti-IL-17 antibody secukinumab significantly reduces signs and symptoms of RA compared with placebo, based on an analysis of biologic-naïve patients (mean disease durations: 6.0 years) [18]. In that clinical study, the RA patients were in the late phase of RA, not the early phase. As mentioned in Section 1, IL-17 plays an important role in the preonset or early-onset phase of the pathogenesis of RA [6, 7]. In addition, in the current study, we showed an elevated ratio of Th17 cell-derived Th1 cells in the early phase of RA, suggesting that Th17 cells are converted to Th1 cells in early RA. Thus, anti-IL-17 antibodies should be used in the preonset or the early-onset phase of RA to obtain more effective therapeutic results. Recently, Schett's group reported the combination of anti-TNF*α* antibodies and anti-IL-17 antibodies at the Fc, which had a measurable effect in a mouse model of RA [19, 20]. Combined antibodies are expected to yield an effective response in patients with early phase RA.

In the current study, MTX, which is “the gold standard” oral medication for RA, significantly reduced the ratio of Th17 cells but not Th1 or Th1∙Th17 cells (Figures 1(a) and 1(b)). In addition, in the 1st group of RA patients, 4 of 5 patients with RA showed improved CRP levels (Table 1). Thus, our findings suggest that Th17 plays an important role in the pathogenesis of early-onset RA. In addition, surprisingly, the ratio of Th1 cells in 3 of 5 patients increased (Figure 1(a) right). These findings may demonstrate the pharmacological effect of MTX; however, they also suggest that Th17 cells are more important than Th1 cells in early-onset patients with RA.

We are now analyzing Th17 cells and Th1 cells using a third group of RA patients with early-onset RA. In the ongoing study, we are identifying Th17 cells and Th1 cells using only cell surface markers, as recommended in the Human Immunology Project of the Human Immunology Study Group [10]. In addition, we are trying to confirm the precision of this method of the Human Immunology Project by measuring the actual productions of cytokines IL-17 and IFN-*γ* and the expression of CD161.

In conclusion, through analyzing the peripheral blood of early-onset RA patients we demonstrated that MTX reduced the ratio of Th17 cells in helper T cells but not that of Th1 cells and that the ratio of Th17 cell-derived Th1 cells to Th17 cells increased. These findings suggest that Th17 cells play an important role in the pathogenesis of early phase RA, indicating the usefulness of anti-IL-17 antibodies in the early phase of RA but not in the late phase of RA or in patients resistant to other biologics such as anti-TNF antibodies.

## 5. Conclusions

In the early-onset RA patients MTX reduced the ratio of Th17 cells among helper T cells but not those of Th1 cells or Th17∙Th1 cells (Th cells producing both IFN-*γ* and IL-17). In addition, the ratio of Th17 cell-derived Th1 cells to Th17 cells increased in the peripheral blood of the early-onset RA patients, compared with those of OA patients.

## Figures and Tables

**Table 1:** Patient profile.

Patient #	Sex	Age (year)	Disease duration (m)	Anti-CCP U/mL	RF		CRP		Treatment MTX mg/w	Duration between 1st and 2nd analysis (m)
					IU/mL		mg/d			
					Pre	Post	Pre	Post		
1	F	30	12	121	1269	96	0.12	0.02	6	6
2	F	63	3	>300	68	56	0.01	0.02	6	1.5
3	F	22	12	>300	327	153	2.70	1.82	4	2
4	M	67	6	251	774	198	2.64	0.36	4	1.5
5	F	40	18	<0.6	6	3	3.66	4.65	4	1

**Table 2:** Profiles of patients.

Patient #	Sex	Age (year)	Diagnosis 2010 ACR/EULAR	Disease duration (m)	CCP U/mL	RF IU/mL	CRP mg/d	Treatment after the analysis
6	F	43	ReA	9	—	—	0.02	—
7	F	57	RA	3	—	—	0.02	MTX 4 mg/w→SASP 500
8	F	71	RA	5	356	923	2.47	Bu 100
9	F	34	RA	1.5	—	—	0.04	—
10	F	51	RA	3	18.9	—	0.01	—
11	F	42	RA	4	280	28	0.09	MTX 6 mg/w
